# Coherent interaction with two-level fluctuators using near field scanning microwave microscopy

**DOI:** 10.1038/srep17176

**Published:** 2015-11-24

**Authors:** S. E. de Graaf, A. V. Danilov, S. E. Kubatkin

**Affiliations:** 1 Chalmers University of Technology, Department of Microtechnology & Nanoscience, MC2. SE-41296 Goteborg, Sweden; 2 National Physical Laboratory, TW11 0LW Hampton Road, Teddington, United Kingdom

## Abstract

Near field Scanning Microwave Microscopy (NSMM) is a scanning probe technique that non-invasively can obtain material properties on the nano-scale at microwave frequencies. While focus has been on developing room-temperature systems it was recently shown that this technique can potentially reach the quantum regime, opening up for applications in materials science and device characterization in solid state quantum information processing. In this paper we theoretically investigate this new regime of NSMM. Specifically we show that interaction between a resonant NSMM probe and certain types of two-level systems become possible when the NSMM probe operates in the (sub-) single photon regime, and we expect a high signal-to-noise ratio if operated under the right conditions. This would allow to detect single atomic material defects with energy splittings in the GHz range with nano-scale resolution, provided that individual defects in the material under study are well enough separated. We estimate that this condition is fulfilled for materials with loss tangents below tan *δ* ∼ 10^−3^ which holds for materials used in today’s quantum circuits and devices where typically tan *δ* < 10^−5^. We also propose several extensions to a resonant NSMM that could improve sensitivity and functionality also for microscopes operating in a high power regime.

Near field Scanning Microwave Microscopy (NSMM) is gaining more and more attention due to its ability to wirelessly and non-invasively extract information about resistivity and susceptibility at the nano-scale^1^,^2^,^3^,^4^. Together with many other scanning probe techniques NSMM is frequently used to obtain information about materials and devices in a wide variety of fields ranging from semiconductor technologies to biological applications^2^,^5^. Here we focus on the application of NSMM to materials commonly used in solid state quantum devices, for which many issues still remain to be solved in order to achieve the necessary reliability needed for future quantum technologies. In particular, material defects and impurities have been shown to constitute a major limitation to achieving long coherence times in these devices^6^. One class of material defects that are of particular importance in solid state quantum devices are so called two-level fluctuators (TLFs). These are atomic-scale defects, present in all dielectrics, which behave as quantum mechanical two-level systems with energy splittings covering a very wide frequency range. Their influence on noise and decoherence have been studied extensively in qubits^6^,^7^ and superconducting resonators^8^,^9^,^10^. The drawback of these methods is that all information about individual defects is not readily obtained: for example, their location can only be determined to within the microwave mode volume. Therefore, in a typical device the microwave field couples to an ensemble of TLFs and, at the same time, mediates the exchange of energy between spatially separated resonant TLFs. Here we show that by using NSMM the microwave electric field can locally be enhanced more than 10^4^ times, which results in much stronger microwave-TLF coupling and allows for localised measurements of single TLFs.

Typically solid state quantum information processing is carried out using microwave photons interacting with a variety of quantum devices^11^, and NSMM naturally lends itself to the task of studying these systems since it operates in the same microwave domain. The class of resonant NSMMs (where the microwave probe is a resonance cavity) have an enhanced sensitivity (the smallest frequency shift that can be detected, determined by, for example, frequency noise of the cavity) compared to non-resonant NSMMs. A NSMM based on a high-Q cavity could be used to gain important information about defects that can lead to improved materials and increased coherence times of solid state quantum devices. However, to reach these conditions several requirements have to be fulfilled. For example, being a two-level system, a single TLF will be saturated by the absorption of a single photon^12^. To avoid saturation the probing cavity should be operated near the single photon regime and at millikelvin temperatures 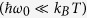.

In addition to TLFs there are several other quantum systems that can be studied, with less stringent requirements on NSMM performance. We take the example of a proposed quantum computing scheme that utilise the spin degree of freedom of intentional material defects to store and manipulate quantum information^13^. For large scale applications these defects must be introduced with very high precision and reproducibility, and in developing the right techniques for this an NSMM could provide valuable feedback. Another application of NSMM can be found in solid state qubit architecture development: Detailed individual qubit characterization could easily be performed using NSMM in order to pinpoint local defective qubits and debug the architecture as well as extracting coherence properties of individual qubits.

In this manuscript we analyse an NSMM operating in the low power limit, where we can treat the problem of probe-sample interaction quantum-mechanically. Our main objective is to determine if single two-level defects can be detected using NSMM, and what the limitations would be. We start by defining our model for tip-TLF interaction and discuss the results in terms of what materials and systems that could be interrogated. This is done first by considering the interaction with a single TLF and then extending the discussion to materials which have a high density of (overlapping) TLF. We discuss the prospects of developing NSMM systems with the requirements for TLF detection based on recent developments^14^ and we propose several modifications of a NSMM setup that could benefit also NSMM operation in the high power limit. Finally we go to a regime where the NSMM probing energy corresponds to a larger number of photons in the cavity and we discuss its application to several other physical systems that could be investigated. As the energy is increased further we enter the classical regime, where the NSMM response has been considered in many different situations, see for example refs 2,3 for reviews.

Based on recent experimental progress we conclude that developing a NSMM for the purpose of detecting individual TLFs is possible and it can be successfully applied to materials where the loss tangent tan *δ* < 10^−3^.

## Model

**Figure 1:**
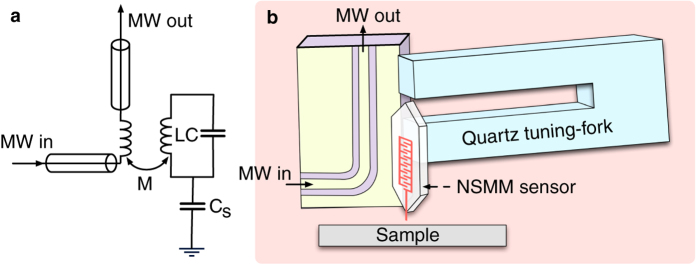
Operating principle of a coherent NSMM discussed in this manuscript. (**a**) Equivalent circuit model for the microwave readout in (**b**). LC represents the microwave cavity which couples to the sample via *C*_*s*_. The cavity is inductively coupled to a transmission line (with mutual inductance M) through which the state of the cavity is read out. (**b**) Setup where the microwave cavity is mounted directly on a quartz tuning fork sensor of a cryogenic AFM.

The general idea behind the NSMM discussed in this paper is depicted in Fig. 1. A high-Q microwave resonator (here we assume a resonance frequency *f*_0_ = 6 GHz) is mounted on a mechanical tuning-fork. The tuning fork is the force sensor used in an atomic force microscope integrated with the NSMM. This allows for precise distance control of the integrated AFM/NSMM scanning probe tip that is brought in close contact (∼nm separation) with the sample under study. The AFM/NSMM tip is maintained at a constant height from the sample surface using a feedback loop that acts on the measured tuning-fork response, and the tip can be moved with subnanomter precision in all three dimensions using piezoelectric transducers. The high-Q microwave resonator that is the core element of the NSMM is probed via the mutual inductance *M* to a mechanically decoupled transmission line nearby. The whole microscope sits in a dilution refrigerator to ensure that the temperature satisfies 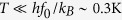. The quantity that is recorded as the NSMM output signal is the frequency shift of the microwave resonator due to the interaction with the sample. Using metrological techniques^15^,^16^ it is possible to measure the frequency shift (and Q-factor) of the resonator with very high accuracy and bandwidth even at single photon energies. A high readout-bandwidth becomes important when using the microwave resonator frequency for scanning probe feedback, which we will discuss later on.

We will now consider theoretically the situation when we have a very low probing amplitude in the resonator such that the average quanta of energy stored is less than the energy *hf*_0_ of a single photon. We can relate the photon number, *N*, to the energy stored in the resonator as 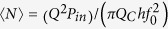
17, where *Q*_*C*_ is the coupling Q-factor and *P*_in_ is the excitation power. First we consider a simplified case without dissipative losses when the NSMM probe is coupled to a generalised TLF described by the uncoupled energy levels *E*_±_ and their difference *E*_+_ − *E*_−_ ≡ *δE*. The levels are coupled via the tunnelling splitting Δ such that the Hamiltonian describing the TLF can be written 
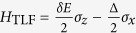
. To illustrate the effect of the TLF on the NSMM probe, we model the system in the so-called dispersive regime. This applies when the resonator is sufficiently detuned from the TLF resonance splitting. The detuning is given by 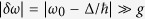, where *g* is the coupling between the microwave field in the resonator and the TLF, *ω*_0_ = 2*πf*_0_, and we assume that the resonance frequency is on the same order as the TLF frequency 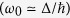. The resonator is modelled as a harmonic oscillator, *H*_*r*_ = *ħω*_0_*a*^†^*a*, and the Hamiltonian describing the coupled system is *H* = *H*_*r*_ + *H*_TLF_ + *H*_coupling_ where *H*_coupling_ = *g*(*a*^†^ + *a*)*σ*_*x*_ is a result of pure electric dipole coupling and no magnetic coupling. The following dispersive Jaynes-Cummings Hamiltonian can then be derived^18^


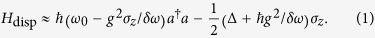


Effectively the resonator acquires a dispersive frequency shift ±*g*^2^/*δω* due to the interaction with the TLF. We also see that the further the TLF is detuned from the resonance frequency of the NSMM probe, the smaller is the induced shift of the NSMM resonance frequency. The above Hamiltonian illustrates the fundamental behaviour of the NSMM probe, however, our detailed model (see methods) takes into account the effect of multiple TLFs and dissipation, and allows us to explore a much wider parameter range in terms of coupling strength and detuning.

**Figure 2:**
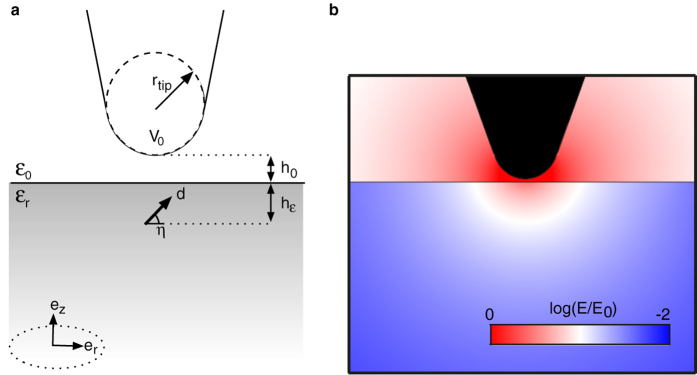
Geometry for the coupling of a TLF to the NSMM probe. (**a**) The TLF is located at a distance *h* = *h*_0_ + *h*_*ε*_ ≪ *r*_tip_ from the apex of the tip. The TLF with dipole moment **d** oriented with an angle *η* with respect to the xy-plane couples to the tip through the induced electric field. (**b**) Electric field strength around the near-field tip and its penetration into a semi-infinite dielectric sample (*ε*_*r*_ = 9).

The coupling strength as a function of the distance between the tip and the TLF is given by


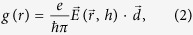


and depends strongly on the electric field 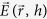 around the tip. To evaluate the electric field we consider the geometry depicted in Fig. 2a: A semi-spherical tip of radius *r*_tip_ is scanned across a flat surface of a homogeneous and isotropic dielectric at a distance *h*_0_. We assume that the thickness of the dielectric is much larger than the tip radius (if the sample instead is a thin dielectric film on top of a metallic surface it will lead to an enhanced electric field strength). A TLF is embedded in the dielectric volume at a distance *h*_*ε*_ from the surface (such that the total distance to the TLF is *h* = *h*_0_ + *h*_*ε*_) with a dipole moment 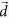 pointing in an arbitrary direction given by the angle *η*. See methods for the exact expression for the electric field in this geometry. For simplicity, in all our calculations below we assume that the TLF dipole is pointing in the z-direction such that the coupling is maximised (*η* = *π*/2). Note that if the TLF dipole is oriented in the plane of the sample (*η* = 0), it will instead couple to the radial component of the electric field from the tip and the observed feature will have a dipole-like appearance rather than radial symmetry, which is the case considered throughout this manuscript. This anisotropy could be used to determine the angle of the TLF dipole and its orientation with respect to the dielectric lattice. We also note that our model takes into account TLF-TLF couplings mediated through the cavity, however, direct TLF-TLF interaction is assumed to be negligible. As the tip is scanned across the surface we change 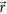, but *h* is assumed to remain constant.

## Results and Discussion

### A single TLF

Single TLF spectroscopy have been demonstrated in Josephson junctions and superconducting resonators incorporating parallel plate capacitors. Here a single TLF can couple strongly via the electric field across the thin parallel plate capacitor. Coupling strengths measure up to several tens of MHz^7^,^19^,^20^. The situation is similar in the case of an NSMM probe: here the electric field is maximised at the near-field tip, and can in principle be of equal strength as in Josephson junctions. However, the geometry and electrostatic environment is different. Using a typical value for the TLF dipole moment *d* ~ 1 eÅ^7^,^21^,^22^, in our model (see methods) we get a maximum coupling between tip and TLF of *g*_*max*_ ≈ 25 MHz. In this estimate we have assumed *h*_0_ = *h*_*ε*_ = 1 nm and *ε*_*r*_ = 9. 25 MHz is a strong coupling strength and should therefore be readily detectable using NSMM, provided that the NSMM operates with a high Q at low temperatures and probing powers and with sufficient mechanical stability. We note that the smaller the tip-TLF separation, the stronger the coupling will be.

**Figure 3:**
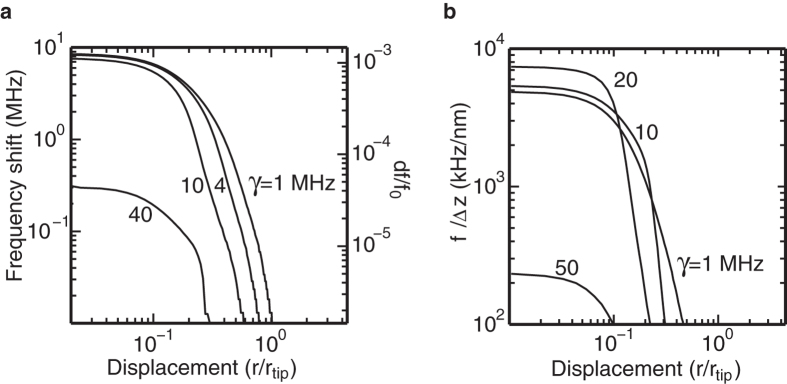
NSMM response to a single TLF. (**a**) Calculated frequency shift of the NSMM probe as a function of its displacement from atop a TLF in Al_2_O_3_ (*h* = *h*_0_ + *h*_*ε*_ = 2 + 1 nm) for several values of the TLF line width *γ* in the dispersive regime. The calculation assumes a TLF detuning *ω*_0_ − *ω*_TLF_ = 2*π* ⋅ 1 MHz, *ω*_0_/2*π* = 5 GHz, *Q* = 50000. (**b**) Change in cavity frequency due to a vertical displacement Δ*z* for the same conditions as in (**a**). We note that the noise floor as obtained in^14^ is below the scale in both (**a**,**b**).

We then turn to the question of what lateral resolution that can be achieved, and if it will be high enough to couple to just a single TLF. In Fig. 3a we have evaluated the frequency shift of the NSMM probe caused by a single TLF. Due to the strong dependence on the electric field the location of the TLF can be determined well beyond the tip size. The parameters used in the calculation in Fig. 3a are typical for the low power NSMM demonstrated in ref. 14, the various TLF decay rates (*γ*) shown correspond to the observed range in aluminium oxide^19^,^23^. The sudden drop in response around *r* = *r*_tip_ in Fig. 3a comes from the transition from the strong to the weak coupling regime, as the tip is moved away from atop the TLF. Our calculated frequency shifts are much greater than the noise levels in state-of-the-art NSMMs^2^,^14^ and should be readily detectable. We here assume that the NSMM utilises fast frequency tracking of the resonance frequency that can capture the full range of frequency shifts obtained here. Such tracking can in particular be implemented using a Pound-locking technique^14^,^15^.

One issue that may prevent detection of TLFs is that the mechanical stability is not good enough: fluctuations in tip sample distance *h*_0_ will directly influence the frequency of the NSMM probe. However, such frequency noise only scales as 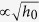 to first order, while the frequency shifts due to a coupled TLF scales as ∝*h*_0_. We therefore propose that even better signal to noise in detecting TLFs using NSMM can be achieved if the NSMM tip-sample separation is oscillating at a fixed frequency, and the NSMM microwave frequency response is read out at the same frequency. Not only does this filter out mechanical noise but a higher measurement frequency will result in less electrical noise. This type of demodulation is particularly suitable in a cryogenic tuning-fork AFM where the distance control is performed by oscillating the cantilever, typically at frequencies of 10’s of kHz. Thus by demodulating the measured NSMM probe frequency due to an oscillating mechanical displacement it is possible to measure the quantity Δ*f*_0_/Δ*z*.

In Fig. 3b we have evaluated the change in microwave frequency as the tip is vertically displaced ±0.25 nm from the static conditions in Fig. 3a. A measurement of the derivative Δ*f*_0_/Δ*z* over a TLF gives an enhanced contrast compared to a Δ*f*_0_/Δ*z* measurement over a metal (or dielectric) surface, which is on the order of 1–10 kHz/nm, i.e well below the scale presented in Fig. 3b. We also note that the TLF line width can be inferred from the line shape of the measured response.

One advantage of operating in the dispersive regime is that the frequency span is not limited by the resonance line width. The frequency shift can be detected (in the case of strong coupling) even when the detuning of the TLF frequency is much bigger than the cavity line width: depending on the TLF coupling, this detection could be feasible for up to a couple of 100 MHz detuning. Furthermore, this regime relaxes the requirements for the photon population of the probe, as will be discussed later. Ultimately there will be a trade-off between the noise floor of the NSMM, the detuning, and the photon population of the probe.

### Dielectrics with a large number of TLFs

We will now consider the situation when the density of TLFs is large enough to cause an overlapping measured response, in order to classify in which type of materials individual TLFs can be studied. We restrict our discussion to the TLFs that can be found in a small volume nearby the tip, since any TLF at a distance of a couple of *r*_tip_ from the tip apex does not significantly contribute to the frequency shift of the NSMM probe.

**Figure 4:**
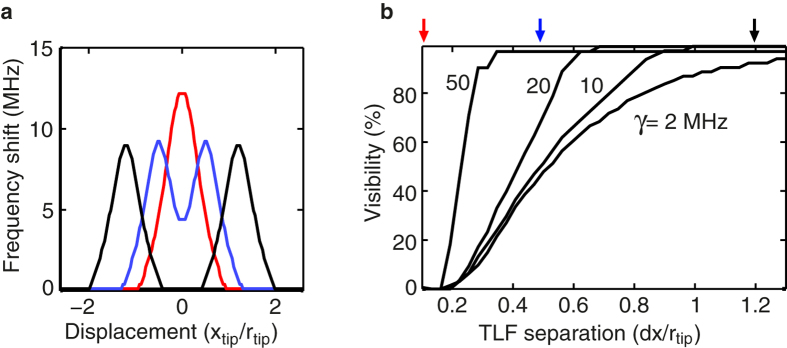
Multiple TLFs. (**a**) The scanned frequency shift profile for three different TLF separations indicated by the coloured arrows in (**b**) (*γ* = 10 MHz). (**b**) Calculated visibility between two adjacent TLFs, separated by a distance *dx*, for several TLF lifetimes. The two TLFs are assumed to be identical, except for their physical location. Other simulation parameters are the same as in Fig. 3.

Consider the response from two identical TLFs placed at locations *x* = ±*dx*/2 as the tip is scanned on top. Figure 4a shows a few examples of such traces calculated using equation (6). To quantify how well two TLFs can be resolved we define the visibility as the maximum frequency shift of the trace divided by the frequency shift at *x* = 0. The result is shown in Fig. 4b for several TLF line widths. Notably, two TLFs may be individually resolved if they are separated down to a distance of about 4 times smaller than the tip radius. For larger separation full contrast is quickly recovered. TLFs with large decay rates (that are weakly coupled) can be distinguished with higher resolution than very long lived TLFs that exhibit stronger coupling, provided that the sensitivity to changes in NSMM resonance frequency is high enough. This is due to the increased dispersive frequency shifts of strongly coupled TLFs, especially at distances away from the centre of the TLF. However, a large *γ* may instead result in a small frequency shift (see Fig. 3a) which falls under the noise floor of the NSMM readout. We note that, accounting for recently demonstrated noise levels^14^, taking into account the noise will only change the results in Fig. 4b by a few % for the largest *γ*, and even less otherwise.

Based on these results we can now proceed to discuss what kind of materials that potentially could be studied in detail using single photon NSMM. We start by making a few assumptions based on the obtained results. First, for a sharp tip (*r*_tip_ < 50 nm) we can limit ourselves to a volume of not more than 100 × 100 × 100 nm as TLFs further away will not couple to the tip. Second, in this small volume there will still exist a large number of TLFs, but only a few of them will have an energy splitting that closely matches the photon energy of the NSMM probe. If we restrict the discussion to only involve TLFs within ±100 MHz of the NSMM frequency (larger differences will produce small dispersive shifts, c.f. equation (1)), we can estimate the response from a wide range of different materials. In order to detect the response from a single TLF we therefore require that, on average, there is less than one TLF in our considered volume and bandwidth.

For example, in aluminium oxide tunnel junctions the TLF density has been measured to be around 0.1 GHz^−1^*μ*m^−2^ 7. This would result in the average of 2 ⋅ 10^−4^ TLFs, with frequencies within 100 MHz of the probe, inside the coupled tip volume, or the tip would have to be scanned over an area of about 50 *μ*m^2^ before finding a TLF with a matching frequency. I.e. the background contribution from adjacent TLFs will be very small in Al_2_O_3_ and similar materials. In general for dielectric materials commonly used in solid state quantum devices we can relate the measured bulk intrinsic loss tangent, tan*δ*, to the density and the dipole moment of TLFs through


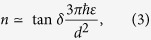


For tan*δ* = 10^−6^ we get *n* ≈ 1 GHz^−1^*μ*m^−3^ assuming atomic scale dipoles. Thus, statistically, there will be more than one TLF in the sample that is coupled to the NSMM probe first when the loss tangent of the material under study exceeds 10^−3^. As an example, recent measurements on silicon nitride thin films^24^ revealed dipole moments of *d* = 1.6 eÅ and a density *n* ≈ 30 GHz^−1^*μ*m^−3^, for a loss tangent of 1.8 × 10^−5^, in good quantitative agreement with the simplified equation (3). Other dielectric materials commonly used in quantum circuits includes sapphire and silicon, both of which has a sufficiently low loss tangent to be studied using this technique. In the presence of direct TLF-TLF interaction^10^,^25^ we would expect our results to be quantitatively similar. The dephasing (which we here assume to be small: *γ*_*ϕ*_ ≪ *γ*) of individual TLFs will result in more broadened line shapes and a reduced visibility. However, the effect on the visibility is expected to be negligibly small for realistic values of *γ*_*ϕ*_.

We then turn to the Q-factor of the probe, which we so far only have assumed to be high. While this is not a strict requirement, our proposed readout technique benefits from increased sensitivity for a higher Q. Furthermore, a high Q will not significantly contribute to the dissipation for the coupled system, highlighting the TLF properties. However, due to the Purcell effect, the NSMM resonator will experience an increased effective decay rate *κ* as a result of a detuned, but coupled TLF. The total decay rate of the resonator when coupled to a TLF is given by *κ* = *κ*_0_ + *κ*_TLF_, where *κ*_0_ = *ω*_0_/*Q* is the bare NSMM resonator decay rate and *κ*_TLF_ = *γ*(*g*/*δω*)^2^ is the Purcell-induced dissipation. We note that even if the sample contains a large number of TLFs (tan*δ* ~ 10^−3^) under the tip volume, only the single TLF within our considered bandwidth of ±100 MHz of *f*_0_ will significantly contribute to a reduced Q of the NSMM probe. On average there will be two TLF with |*δω*/2*π*| = 100–300 MHz, two with |*δω*/2*π*| = 300–500 MHz and so on. Together they will lead to a decay rate that only depends on the TLFs that are close in frequency: 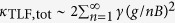, where *B* = 200 MHz is the bandwidth we consider in the above example.

### Prospects for developing coherent NSMM systems

A large number of NSMMs have been demonstrated in the high power (classical) regime and they usually combine microwave readout with either scanning tunnelling microscopy (STM) or atomic force microscopy (AFM) to achieve accurate tip-sample distance control^1^,^26^,^27^,^28^, which is crucial for coherent interaction since the coupling depends strongly on tip-sample separation. The microwave response is then read out simultaneously as the topography is recorded. While this technique greatly suffers from cross talk from the sample topography in the microwave image^29^ another option is to use the response of the microwave resonator itself as the feedback signal to follow the sample topography^14^. This method can be used to separate the microwave response from the topography, given that the topography is already known. Passively recording the microwave response while distance control is maintained by AFM feedback in principle allows for very long acquisition times, and therefore the photon number of the resonator can be significantly reduced at the expense of averaging time. Pure NSMM feedback still has to maintain a high bandwidth not to crash the tip in the presence of, for example, mechanical vibrations, which makes it more challenging to reach the single photon regime. Successful operation in pure NSMM mode with a photon population 〈*N*〉 = 1000 and a bandwidth of 150 Hz was recently demonstrated^14^. The obtained sensitivity to frequency shifts (*δf*/*f*_0_ ≈ 4 ⋅ 10^−8^ limited by mechanical noise and *δf*/*f*_0_ ≈ 2 ⋅ 10^−7^ for *N* = 1000) was much higher than the frequency shifts due to TLFs discussed above. Using state-of-the-art cryogenic amplifiers the photon number from this experiment could be reduced significantly while maintaining the same bandwidth and sensitivity. Further improvement could be achieved by using a (near) quantum-limited parametric amplifier^30^. Other NSMM cavity designs such as coaxial resonators^31^ or dielectric resonators^32^ could potentially be used to achieve the same goals. The main shortcoming of the NSMM in ref. 14 was the high operating temperature of 300 mK, while achieving lower operation temperatures of scanning probe systems can be challenging, it is certainly possible^5^,^33^. Reaching the requirements discussed in this manuscript for a NSMM that is capable of interacting with individual TLFs is therefore mainly a technical challenge, where all individual requirements already have been demonstrated. This includes nano scale NSMM resolution^14^,^26^, high precision measurements of superconducting resonators below single photon populations^10^, on-chip coupling via the near-field to macroscopic quantum devices^11^ and individual TLFs in qubits^7^,^12^,^19^ and in superconducting resonators^20^.

To further improve the capabilities of a coherent NSMM to study TLFs it would useful to have the ability to tune the frequency of the resonant NSMM probe. Tunable superconducting resonators are frequently used in solid state quantum circuits, for example to shift a resonator in and out of resonance with qubit(s)^34^. This is typically realised by using the non-linear inductance of a superconducting quantum interference device (SQUID), which can be rapidly tuned with applied magnetic flux^35^. Alternatively, in the search for TLFs, their splitting can in some cases be controlled by an external DC electric field 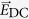
20. The static field polarises the dielectric environment and changes the TLF transition energy according to 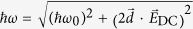. The required fields are on the order of 10–100 kV/m. In the NSMM configuration, such fields translates to relatively small tip bias voltages, which noninvasively can be implemented into superconducting resonators^14^,^36^. A NSMM incorporating voltage bias of the tip and/or resonance frequency tuning would thus be able to obtain valuable information about the frequency *and* spatial distribution of individual TLFs.

### NSMM in the many-photon regime

Whereas constructing a single photon NSMM may seem like a large undertaking, useful information about different quantum systems may also be obtained in a different regime, where the probing power in the NSMM is increased by several orders of magnitude. While the above analysis is only valid for very low photon numbers *N*, a single photon (〈*N*〉 < 1) population is not a strict requirement for observing TLFs using NSMM. Saturation occurs once the steady state population of the excited state *P*_*e*_ of the TLF approaches 1/2 due to the energy transferred from the cavity, and to avoid saturation the following requirement must be fulfilled^37^:


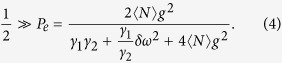


In the case of strong coupling (*g* ≫ *γ*_1_) and large detuning (*δω* ≫ *g*) we can approximate the number of photons that lead to saturation to 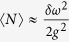, which increases quadratically with detuning, while the frequency shift of the NSMM probe decreases only linearly to first approximation. We thus note that it could be feasible to detect TLFs using large dispersive detuning even with photon numbers up to 〈*N*〉 = 10^2^–10^3^, depending on the parameters of the system. Whether this regime is reachable using NSMM remains to be seen, since it also strongly depends on the decoherence properties of the individual TLF.

In the same way as TLFs can be studied so can macroscopic, artificially made qubits. Due to their much larger size the coupling, *g*, will mainly depend on the tip size and its separation to the surface, and could therefore be precisely controlled over a very wide range. Thus, a NSMM populated with a larger number of photons could dispersively read out the properties of solid state qubits with a higher signal to noise ratio than what is expected for a single TLF, and application of an NSMM to study large arrays of superconducting qubits in an integrated circuit could become a useful tool in the development of better quantum circuits.

Another relevant application of the NSMM could potentially be within defect-based quantum computing. Here the ultimate goal is to use the spin degree of freedom of a single, artificially created, defect to store and manipulate quantum information, and high-Q superconducting resonators have been proven to be highly sensitive for interacting with small ensembles of molecular spins^16^,^34^,^38^ and they can be made robust enough to withstand the required magnetic fields^36^,^39^. While this manipulation mainly relies on coupling to the magnetic component of the microwave field, scalable quantum technologies will require that manipulation can be performed via electric fields, since these can be localised on much smaller length-scales. The development of large scale technologies would require techniques that very precisely introduce a well defined number of defects at certain locations in a substrate, which would greatly benefit from nano scale imaging. Our estimations show that an NSMM can only be applied to materials where the spin-defects are subjected to strong polar environments, or where significant spin-orbit interaction is present. An example of such a system is colour centres in silicon carbide^40^. We estimate that a single colour centre at a distance of 1 nm would couple to the NSMM probe with strength on the order of *g* = 2 kHz. In strongly piezoelectric materials, such as zinc oxide, the coupling could be an order of magnitude stronger^41^,^42^. Furthermore, this electric coupling can collectively be enhanced by a factor 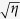, where *η* is the number of defects in an ensemble under the NSMM tip. In this situation the NSMM probing power can be increased even further to obtain a better signal to noise ratio since saturation of the whole spin ensemble will occur at much higher ensemble excitation energies.

## Methods

The Hamiltonian used in our calculations has the following form in the rotating frame of an input drive signal of amplitude A and frequency *ω*_*d*_:


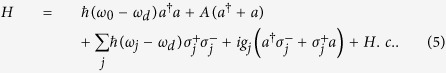


*j* sums over all TLFs. We model the time-evolution of the coupled system with the master equation 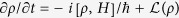, and a Lindblad operator on the form 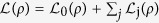, where


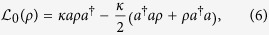



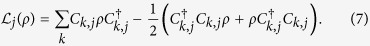


Here *κ* = *ω*_0_/*Q* is the cavity decay rate and the operators 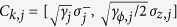 accounts for intrinsic TLF energy relaxation and pure dephasing of the TLF respectively. The decoherence rate *γ*_2_ relates to the relaxation rate and the pure dephasing rate as *γ*_2_ = *γ*/2 + *γ*_*ϕ*_. The frequency shift of the NSMM probe


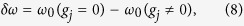


is evaluated with a coupling that depends on the electric field around the NSMM tip, *g*_*j*_ = *g*_*j*_(*E*(*r*_*j*_)), and *ω*_0_(*g*_*j*_ ≠ 0) is the angular frequency at which the spectral output from the coupled NSMM resonator - TLF system, 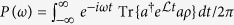, is maximised.

The electric field from the tip onto the TLF as given by the geometry in Fig. 2a. The electric field has the following form^43^


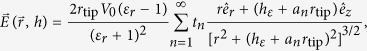


with recursive coefficients *a*_1_ = (*r*_tip_ + *h*_0_)/*r*_tip_, *a*_*n*_ = *a*_1_ − 1/(*a*_1_ + *a*_*n*−1_), *t*_1_ = 1, and *t*_*n*_ = *t*_*n*−1_/(*a*_1_ + *a*_*n*−1_). The voltage on the tip is given by the microwave voltage amplitude generated by a single photon in the cavity, 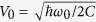.

All calculations are performed using the quantum optics toolbox for Matlab^44^.

## Additional Information

**How to cite this article**: Graaf, S. E. *et al.* Coherent interaction with two-level fluctuators using near field scanning microwave microscopy. *Sci. Rep.* **5**, 17176; doi: 10.1038/srep17176 (2015).
